# Efficacy of a Mobile Health-Supported Home-Based Resistance Exercise After Ultrasound-Guided Corticosteroid Injection in Chronic Subacromial Bursitis: A Randomized Controlled Trial

**DOI:** 10.3390/jcm15072567

**Published:** 2026-03-27

**Authors:** Yuan-Chen Chang, Ming-Ta Yang, Yu-Hsuan Cheng, Chun-De Liao, Kwang-Hwa Chang, Pei-Chun Wong, Shih-Wei Huang

**Affiliations:** 1 Department of Physical Medicine and Rehabilitation, Wan Fang Hospital, Taipei Medical University, Taipei 116, Taiwan; 112131@w.tmu.edu.tw (Y.-C.C.); 105168@w.tmu.edu.tw (Y.-H.C.); 94189@w.tmu.edu.tw (K.-H.C.); 2 Center for General Education, Taipei Medical University, Taipei 11031, Taiwan; yangrugby@tmu.edu.tw; 3 Clinical Research Center, Taipei Medical University Hospital, Taipei 11031, Taiwan; 4 Department of Physical Medicine and Rehabilitation, School of Medicine, College of Medicine, Taipei Medical University, Taipei 11031, Taiwan; 5 Department of Physical Medicine and Rehabilitation, Shuang Ho Hospital, Taipei Medical University, New Taipei City 235, Taiwan; chundeliao@tmu.edu.tw; 6 Master’s Program in Long-Term Care, College of Nursing, Taipei Medical University, Taipei 11031, Taiwan; 7 Graduate Institute of Biomedical Optomechatronics, College of Biomedical Engineering, Taipei Medical University, Taipei 11031, Taiwan

**Keywords:** subacromial impingement syndrome, subacromial bursitis, corticosteroid injection, telerehabilitation, mHealth, instant messaging

## Abstract

**Background**: Corticosteroid injections provide short-term relief for chronic subacromial bursitis but are associated with high recurrence rates. This study investigates the efficacy of a mobile health-supported home-based resistance exercise program compared with exercise education in patients with chronic recurrent subacromial bursitis after ultrasound-guided corticosteroid injections. **Methods**: Participants with chronic subacromial bursitis were assigned via computer-generated block randomization to either an intervention group receiving ultrasound-guided corticosteroid injections followed by a 12-week home-based exercise program (50 min strengthening and resistance/session, 5 days per week) supported via instant messaging applications, or a control group receiving the same injection followed by printed educational materials covering the same exercise protocol. Shoulder Pain and Disability Index (SPADI) scores, Visual Analog Scale (VAS) pain scores and active pain-free range of motion (ROM) were evaluated by a blinded assessor at weeks 4 and 12. Between-group comparisons were analyzed using two-way ANOVA after confirming normality and homoscedasticity. **Results**: Fifty-three patients (mean age: 55.6 ± 10.5 years; 47.2% female) were randomized to the intervention (*n* = 27) or control (*n* = 26) groups. Significant interaction effects were identified for SPADI (*p* = 0.040) and ROM (abduction: *p* = 0.036/ flexion: *p* = 0.032). Post hoc analysis revealed that the intervention group exhibited a significantly greater reduction in SPADI scores (*p* = 0.007, *d* = 0.72) and greater increase in abduction ROM (*p* = 0.004, *d* = 0.84) at 12 weeks; both gains surpassed the MCID. **Conclusions**: A mobile health-supported home-based resistance exercise program can significantly extend the benefits of corticosteroid injections in patients with chronic subacromial bursitis. Trial Registration: NCT06220643, registered 14 December 2023.

## 1. Introduction

Subacromial impingement syndrome is characterized by pain during shoulder abduction or overhead activities, which typically arises from mechanical impingement within the subacromial space or inflammation of the surrounding tissues [1]. Subacromial bursitis represents a major component of this spectrum in which diagnosis is commonly based on ultrasonographic evidence of bursal thickening exceeding 2 mm and a positive response to diagnostic subacromial anesthetic injection [2,3]. Chronic subacromial bursitis often leads to persistent shoulder pain without spontaneous recovery. While initial management involves activity modification and physical therapy, subacromial corticosteroid injections are frequently employed for persistent cases to alleviate pain and facilitate rehabilitation [4]. Ultrasound guidance enhances delivery precision, with corticosteroids combined with local anesthetics as injectate [5]. However, the benefits of injection are often transient. Approximately 40% of patients require repeat injections within three months of initial treatment [6], highlighting the challenge of maintaining long-term symptom relief in chronic cases.

Recurrence is associated with biomechanical factors, including scapular dyskinesis and improper posture due to muscular imbalances around the shoulder that exaggerate impingement [7]. While exercises targeting scapular stabilization can restore proper movement patterns [7,8], these exercises are technically nuanced and often difficult for patients to perform correctly without supervision [9]. Recent randomized controlled trials demonstrated that structured exercise programs can prolong the therapeutic effects of corticosteroid injections in managing chronic subacromial bursitis [10,11]. However, these studies relied primarily on clinic-based physiotherapy, which is often limited by cost, transportation barriers, and scheduling constraints. Consequently, how to effectively integrate exercise after injection to sustain post-injection benefits remains unclear.

Home-based exercise is a feasible alternative [12]. However, the necessity of supervision remains controversial [13]. Large pragmatic trials offered inconsistent evidence in broader shoulder disorders. One large, randomized trial reported no added benefit of supervised progressive exercise over a single best-practice advice session [14], while another study demonstrated the superiority of supervised exercise at 6 months, although the advantage diminished at 12 months [15]. Importantly, these studies enrolled heterogeneous populations rather than specifically chronic subacromial bursitis patients. In patients with chronic recurrent bursitis, unsupervised exercise based on initial education alone (e.g., single exercise instructions with an educational leaflet) may be insufficient to ensure the exercise quality and adherence necessary to prevent recurrence.

Over the past five years, telerehabilitation has experienced unprecedented growth, primarily accelerated by the COVID-19 pandemic, which necessitated a paradigm shift in the delivery of rehabilitative care [16]. Beyond ensuring continuity of service, telerehabilitation has demonstrated significant clinical benefits, including improved patient accessibility, reduced travel burden, and high cost-effectiveness, with outcomes comparable to traditional in-person therapy for musculoskeletal conditions [17]. Telerehabilitation can be categorized into synchronous (e.g., real-time video) and asynchronous (e.g., messaging and apps) modalities. While previous research has predominantly focused on synchronous modalities [18], asynchronous mobile health (mHealth) interventions using everyday technologies (e.g., instant messaging) have emerged as a promising, flexible alternative [19]. However, a recent scoping review [20] on mobile messaging for musculoskeletal pain conditions revealed that only one focused on shoulder disease [21], and no study has examined whether an mHealth-supported intervention provides additional benefits over exercise education specifically in preventing recurrence following corticosteroid injection for chronic subacromial bursitis. This knowledge gap is clinically meaningful, as many patients rely on injections for short-term relief but lack accessible strategies to maintain therapeutic benefits. Therefore, this study aimed to investigate the efficacy of an mHealth-supported home-based resistance exercise program compared with exercise education in patients with chronic recurrent subacromial bursitis after ultrasound-guided corticosteroid injections.

## 2. Materials and Methods

### 2.1. Study Design

This randomized controlled trial was conducted at a medical university hospital. Participants provided written informed consent after receiving a detailed explanation of the study aim and procedures. A research assistant used computer-generated random numbers with a block randomization method (block size of 4) to randomize participants into groups. Group allocation was performed with sealed envelopes. A physician blinded to group allocation performed the ultrasound-guided subacromial bursa injection. The assessor who measured outcomes remained blinded to group allocation throughout the study. The study was approved by the Taipei Medical University Joint institutional review board (IRB No. N202306022) on 20 October 2023, and registered at clinicaltrials.gov (NCT06220643) on 13 December 2023. This study was conducted in accordance with the Declaration of Helsinki, complied with all relevant regulations, and followed the CONSORT guidelines [22].

### 2.2. Participants

Patients with chronic subacromial bursitis were recruited from the outpatient department between 31 January 2024, and June 2024. After initial screening based on patients’ history and physical examination in clinics, an ultrasound-guided screening was performed to identify potential eligible cases.

The inclusion criteria were as follows: (1) age ≥ 50 years (to focus on chronic degenerative conditions and exclude acute sports injuries, aligning with our aging-population research initiative), (2) previous corticosteroid injection for subacromial bursitis > 6 months ago, (3) recurring shoulder pain within the past 6 months, (4) positive Neer’s or Hawkins’ tests for shoulder impingement, (5) painful arc during shoulder abduction or internal rotation, and (6) history of immediate pain relief following an ultrasound-guided injection of corticosteroid mixed with lidocaine (2 mL of 1% lidocaine) into the subacromial bursa.

The exclusion criteria were as follows: (1) receipt of steroid, platelet rich plasma, or hyaluronic acid injections in the affected shoulder within the past 6 months, (2) surgery history on the affected shoulder, (3) rotator cuff tear or calcific tendonitis, (4) shoulder joint or capsule lesions, and (5) neurological disorders affecting cognitive function or pain sensation. Baseline data on basic characteristics (age, sex, body mass index [BMI], affected side, and pain duration) were collected at enrollment.

Participants were withdrawn from the study based on the following criteria: (1) voluntary withdrawal of consent; (2) development of new shoulder pathologies or receiving additional invasive treatments (e.g., surgery or alternative injections) on the affected side during the 12-week study period; or (3) occurrence of severe adverse events. Data analysis was performed following the per-protocol principle.

Regarding pharmacological treatment, participants were allowed to use NSAIDs or acetaminophen for rescue analgesia if necessary. However, the use of oral corticosteroids, opioids, or other invasive shoulder treatments was prohibited throughout the 12-week study period.

### 2.3. Ultrasound-Guided Corticosteroid Injection

A physician, blinded to group allocation and the injectant, administered ultrasound-guided injections into the subacromial bursa using a Philips Affiniti 50 system equipped with a 5–12-MHz linear array transducer. The system’s accuracy for guided subacromial injections and its precision for measurements of fine anatomical structures are well-supported by literature [23,24]. Patients were seated with their arms extended and their hands placed on the buttock of the affected side (i.e., modified Crass position). The transducer was placed on the short axis of the supraspinatus tendon adjacent to the rotator cuff interval. The needle was inserted following a lateral-to-medial trajectory through the deltoid muscle and supraspinatus tendon to access the subacromial bursa. Under ultrasound guidance, the patients received an injection of 1 mL of 40 mg of triamcinolone acetonide mixed with 2 mL of 1% lidocaine, administered using a 23-gauge, 1.5-inch needle. After injection, every patient was monitored for 20 min for possible complications and adverse effects were recorded during follow-up period.

### 2.4. Mobile Health-Supported Home-Based Resistance Exercise

**Figure 1:**
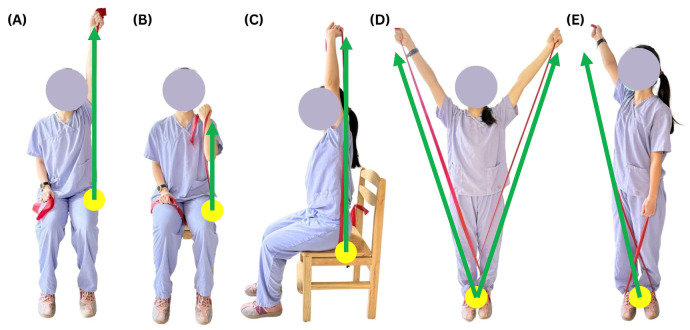
Home-based elastic band resistance exercises for strengthening the shoulder muscles. Yellow dots indicate anchoring points, and green arrows indicate movement trajectories. (**A**) Seated Unilateral Shoulder Abduction: The band is anchored under the thigh; the arm is raised overhead in the frontal plane, targeting the deltoid and supraspinatus. (**B**) Seated Biceps Curl: The band is held under the thigh; the elbow is flexed toward the shoulder to train the biceps brachii and brachialis. (**C**) Seated Overhead Triceps Extension: The band is anchored behind the back (e.g., under the glutes or chair); the elbow is extended upward, targeting the triceps. (**D**) Standing Bilateral Scaption: The band are anchored under the feet; arms are raised diagonally at scapular plane, focusing on the supraspinatus and teres minor. (**E**) Standing Diagonal Shoulder Abduction: Similar to scaption, uses a cross-body pull to further isolate the supraspinatus and teres minor.

The participants in the mHealth-supported exercise group began using Thera bands three days after receiving the ultrasound-guided corticosteroid injection. To ensure safety and exercise quality, the program was facilitated through a smartphone-based instant messaging application (Line; Line Corp, Tokyo, Japan). This platform was selected for its high penetration rate in Taiwan and user familiarity, which effectively minimized technical barriers for participants. Participants engaged with the platform via multimodal communication, including receiving daily reminders for each session and utilizing asynchronous video submissions or text inquiries regarding their exercises. A senior licensed physical therapist provided clinical oversight through responsive feedback to adjust performance for subsequent sessions. The exercise protocol and dosage were synthesized from evidence-based guidelines [25] and adapted from recent clinical trials utilizing elastic bands for subacromial bursitis [10]. The intervention lasted 12 weeks, with five sessions per week. Each session consisted of two parts: 10 min of scapular stabilization exercises and 40 min of elastic band resistance exercises. Following the biomechanical principle of prioritizing scapular control before rotator cuff mobilization, scapular stabilization exercises (including retraction and wall slides) were performed first without an elastic band to provide a stable base and optimize the subacromial space. For the elastic band resistance exercise, we utilized a standardized exercise protocol to train shoulder muscle groups including deltoid, supraspinatus, teres minor, biceps brachii, brachialis, and triceps (Figure 1). Participants performed 3 sets of 10 repetitions of gentle concentric and eccentric contractions through maximal tolerated ROM for each arm. All participants began with a yellow band, which offered the lowest resistance. After each set, perceived exertion was assessed using a visual scale of 0–10. If perceived exertion ratings of ≤6, five additional repetitions were added, up to a maximum of 20. Once 20 repetitions were achieved, participants could increase resistance progressively by advancing to the next color band in sequence (yellow, red, green, blue, black, or silver, an increase in resistance of 20% with each color) in the subsequent session.

### 2.5. Exercise Education Group

After receiving the ultrasound-guided corticosteroid injection, participants in the exercise education group received a 20-min individualized educational session by a senior physical therapist with a booklet containing exercises instructions and visual illustrations identical to the exercises provided to the home exercise group. They were instructed to perform these exercises independently at home, starting three days post-injection, without the support of digital reminders or remote supervision. To ensure their safety, they visited the laboratory for data collection at the designated time points and recorded any adverse events throughout the study period.

### 2.6. Outcome Assessment

The primary outcome measures were Shoulder Pain and Disability Index (SPADI) and Visual Analogue Scale (VAS) scores. The secondary outcome measure was active ROM. SPADI is a self-reported questionnaire comprising 13 items that assess shoulder pain and disability. The total SPADI score ranges from 0 to 100, with higher scores indicating greater pain and disability [26]. We utilized the validated Chinese version of the SPADI, which has demonstrated excellent internal consistency (Cronbach’s α = 0.91) and test–retest reliability (ICC = 0.89) [27]. According to previous research, a change of 18 points in the SPADI score is considered minimal clinically important difference (MCID) [28]. The VAS was used to assess the most intense shoulder pain experienced during the past week on a 0–10 scale, with 0 representing no pain and 10 representing extreme pain [29]. It is a validated instrument with excellent test–retest reliability (ICC = 0.97) in pain measurement [30,31]. Active ROM of the affected shoulder was assessed with a standard goniometer to determine the pain-free limit for flexion, abduction, external rotation, and internal rotation. Each measurement was repeated three times, and the maximal value was recorded. To evaluate clinical relevance, improvements were compared against established MCID thresholds: 12° for forward flexion, 7° for abduction, and 3° for external rotation [32]. All measurements were taken at baseline, and at weeks 4 and 12. Compliance rate was calculated based on the number of participants who visited the laboratory for data collection at the designated time points in each group. Recurrence was referred to as a new onset of moderate shoulder pain lasting more than 1 day following a period of at least 1 week of being pain free (pain intensity 0 or 1) or as an episode of care seeking for the same problem [11]. Recurrence rates were recorded for each group at week 12.

### 2.7. Sample Size

A power analysis using G*Power (version 3.1.9.2) was used to determine the sample size required to detect between-group differences in VAS scores at 12 weeks postinjection. An independent *t* test was specified, targeting a power of 0.80 and α = 0.05. Based on a previous study by Zhu et al. [10], and considering a type I error of 0.05, a power of 80%, and a two-sided test, at least 23 participants per group were required [33].

### 2.8. Statistical Analysis

Demographic and baseline characteristics for each group were analyzed using descriptive statistics. Results for continuous variables are presented as means and standard deviations, and those for non-normally distributed variables are presented as medians and percentiles. Results for categorical variables are presented as frequencies and percentages. Baseline comparisons between groups were performed using independent *t* tests for continuous data and chi-squared or Fisher’s exact tests for categorical data. Before analysis, parametric assumptions were verified using the Shapiro–Wilk test (normality) and Levene’s test (homoscedasticity). A two-way ANOVA was employed to assess the effects of Time, Group, and their Interaction. Significant effects were further explored using Tukey’s post hoc tests for inter-group comparisons at each time point. Effect sizes were reported as partial eta-squared (η**_p_^2^**) for ANOVA and Cohen’s d for post hoc comparisons. According to standard criteria [34], the magnitude of partial eta-squared (η**_p_^2^**) was interpreted as small (0.01), medium (0.06), or large (0.14). Cohen’s d thresholds were based on empirical data for kinesiotherapy [35]: small (0.1), medium (0.3), and large (0.7). All statistical analyses were performed using SPSS version 25.0, with a two-tailed *p* value of <0.05 indicating statistical significance.

## 3. Results

**Figure 2:**
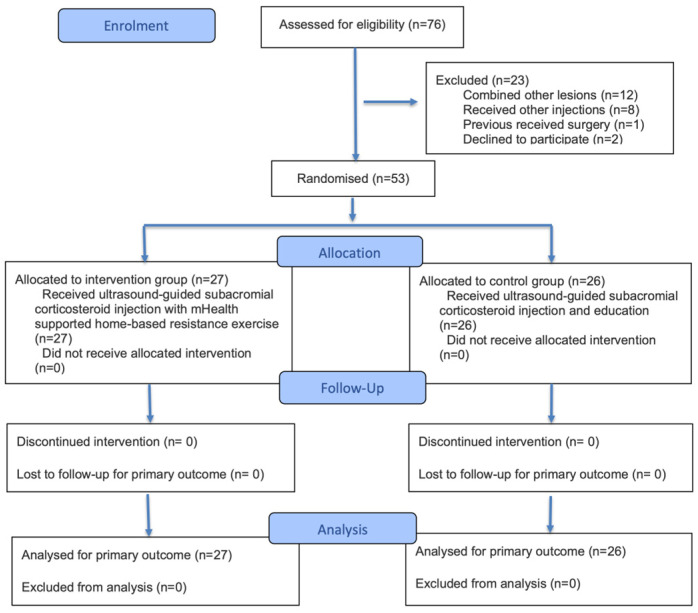
Study flowchart.

A total of 76 patients were screened for eligibility, of which, 23 were excluded, resulting in a study population of 53 participants (27 in the mHealth-supported home exercise group and 26 in the exercise education group; Figure 2).

The demographic and baseline characteristics of the participants are summarized in Table 1. No significant differences were observed between the ultrasound-guided corticosteroid injection with mHealth-supported resistance exercise (UCI + RE) group and the ultrasound-guided corticosteroid injection with exercise education (UCI) group in terms of age, sex, BMI, employment status, affected side and symptom duration. These results indicate that the baseline characteristics were comparable between the two groups.

Prior to the analysis, all data were evaluated for the assumptions required for parametric testing. The Shapiro–Wilk test confirmed that the primary outcomes followed a normal distribution (*p* > 0.05). Homoscedasticity was verified across all groups using Levene’s test, with no significant differences in variances observed for any primary or secondary parameters (all *p* > 0.05).

Primary outcomes, comprising VAS and SPADI scores at baseline and at 4 and 12 weeks postintervention for both groups, are summarized in Table 2. For the VAS score, the two-way ANOVA revealed a significant Time effect (F(2102) = 167.31, *p* < 0.001, η_p_^2^ = 0.77, indicating a large effect), but no significant Interaction effect (*p* = 0.898, η_p_^2^ = 0.002). Although both groups showed significant reductions in pain from baseline to week 4 and week 12 (all *p* < 0.001), there were no significant between-group differences at any time point (all *p* > 0.05). Regarding the SPADI score, a significant Interaction effect (Time × Group) was identified (F(2102) = 3.32, *p* = 0.040, η_p_^2^ = 0.061, medium effect), along with a significant Time effect (*p* < 0.001, η_p_^2^ = 0.773, large effect). Post hoc comparisons revealed that both groups improved significantly from baseline. Notably, the UCI + RE group demonstrated a significantly greater reduction in SPADI scores than the UCI group at week 12 (mean difference, −6.08; 95% CI: −10.46 to −1.70; *p* = 0.007), exhibiting a medium-to-large effect size (Cohen’s *d* = −0.77).

Secondary outcomes, specifically the active pain-free ROM, are detailed in Table 3. Two-way ANOVA revealed significant Interaction effects for Abduction (*p* = 0.036, η_p_^2^ = 0.063) and Flexion (*p* = 0.032, η_p_^2^ = 0.065), both representing medium effect sizes. Additionally, significant Time effects were found across all ROM parameters (all *p* < 0.01). Post hoc analyses revealed no significant between-group differences at week 4 (all *p* > 0.05). However, at week 12, the UCI + RE group showed significantly greater improvements compared to the UCI group in Abduction (*p* = 0.004, *d* = 0.84, large effect) and Flexion (*p* = 0.017, *d* = 0.68, medium effect). In contrast, no significant interaction effects were found for external or internal rotation (all *p* > 0.05).

All participants in the UCI + RE group attended every session through instant messaging, and all participants in the UCI group returned for follow-up assessments at the designated time points. No adverse events or complications from the corticosteroid injection or exercises were observed in either group. No participants reported symptom recurrence during the study.

## 4. Discussion

Our study demonstrated that an mHealth-supported home-based resistance exercise program led to a significant reduction in shoulder pain and disability, and an increase in flexion and abduction active pain-free ROM by week 12. The significant interaction between time and treatment group suggests that beyond the pain relief provided by corticosteroid injections, adding mHealth-supported home exercise creates a more favorable recovery trajectory for shoulder function and mobility compared to the exercise education group. This combined approach may also reduce reliance on repeated injections and help prevent symptom recurrence in patients with chronic subacromial bursitis and impingement syndrome.

The superiority of the mHealth-supported exercise group at 12 weeks highlights the critical role of technology-enabled clinical oversight in rehabilitation. Although previous meta-analyses and randomized controlled trials have indicated that supervised training is no more effective than self-training [13,36], a critical distinction in this study lies in the patient population. Most previous trials enrolled heterogeneous groups; however, our study focused specifically on chronic recurrent subacromial bursitis patients. Patients with long-standing symptoms often develop entrenched maladaptive movement patterns, such as scapular dyskinesis and upper trapezius dominance, as compensatory strategies to avoid pain [37,38]. For this specific cohort, simple repetition of exercises without correction may inadvertently reinforce these compensatory strategies rather than resolve them. Therefore, to effectively prevent recurrence, the success of therapeutic exercises depends fundamentally on exercise accuracy (quality) and adherence (quantity).

Regarding quality, scapular stabilization must be performed with high precision to restore proper neuromuscular control. Without professional feedback, even patients in the exercise education group who adhere to the program might fail to correct these kinematic faults. Beyond movement quality, the instant messaging platform introduced a critical mechanism of accountability. In contrast to unmonitored home exercises where nonadherence rates often range between 30% and 50% [39], the ongoing interaction via the messaging app created a transparent digital footprint. This digital interaction also fosters a robust therapeutic alliance, which is a known predictor of clinical success [40], particularly for individuals with chronic musculoskeletal pain [41]. Prior evidence has demonstrated that higher ratings of the therapeutic alliance increase the likelihood of patients reaching the MCID in pain and functional outcomes during musculoskeletal rehabilitation [42]. The mHealth platform facilitates this through patient-centered interaction, characterized by timely feedback and sensitivity to emotional concerns, which are key communication factors known to strengthen the therapeutic bond [43]. By ensuring that patients feel ‘heard’ and ‘guided’ despite the lack of physical proximity, this integrated structure effectively preserves the human element of rehabilitation in a virtual environment. Therefore, our findings suggest that continuous feedback provided by assistive technology is beneficial for managing this chronic recurrent condition.

This study establishes that this mHealth-supported exercise can effectively serve as a surrogate for face-to-face physiotherapy. Currently, telerehabilitation has evolved beyond a crisis-response tool into a sustainable model for ensuring equitable access to musculoskeletal rehabilitation, particularly for populations facing geographical or mobility barriers [44,45]. The primary advantages include increased convenience, cost-effectiveness, and the integration of rehabilitation into the patient’s daily environment, which promotes long-term behavioral change [46,47]. Despite these advantages, certain limitations persist, such as the inability to perform hands-on manual therapy, potential technical challenges for older populations, and the need for patients to possess a baseline level of health literacy and digital competence [48,49]. Beyond these general considerations, evidence of telerehabilitation targeting shoulder disorders remains limited, as previous research has often focused broadly on rotator cuff tendinopathy or general musculoskeletal conditions. A recent meta-analysis on non-operative shoulder disorders indicates that telerehabilitation over 12 weeks is comparable to in-person rehabilitation for reducing pain, whereas shorter durations showed limited effectiveness [50]. Notably, it only included three trials targeting subacromial impingement syndrome, each with heterogeneous intervention protocols and none incorporating corticosteroid injection [51,52,53]. Similarly, another systematic review reported, based on low-certainty evidence, that telerehabilitation is not superior to in-person physical therapy or home-based exercise programs in improving shoulder pain and disability [54]. Our findings extend this evidence base to chronic subacromial bursitis: the significant Group × Time interaction underscores the importance of a sustained longer-duration mHealth-supported intervention to achieve meaningful pain reduction.

This study provides valuable insights regarding the optimal timing for initiating exercise following corticosteroid injection. Although exercise was initiated 3 days post-injection in our protocol, significant between-group differences were not observed until week 12. This delayed divergence aligns with the pharmacokinetic profile of triamcinolone acetonide, which has a reported biological half-life of approximately 213 h (about 8.9 days) [55]. During the first 4 weeks, which represents roughly 3.1 half-lives, the potent analgesic effect of the corticosteroid likely masked the additive benefits of exercise, creating a ceiling effect where exercise added little observable symptomatic relief initially. However, as pharmacological efficacy typically diminishes after 4 weeks [56], the therapeutic advantage of exercise became evident. These findings are consistent with previous trials [10], where the superiority of combined therapy only became apparent at 12–24 weeks. This relationship highlights that the primary role of the injection is to provide a pain-free window for exercise initiation, while the sustained therapeutic benefit relies on the long-term effects of the mHealth-supported exercise program.

This study has several limitations. First, the adherence and actual exercise volume were not objectively measured in the control group. While this reflects the pragmatic aspect of clinical practice, where objective monitoring of home programs is often not feasible, the lack of absolute training volume in the control group precludes a precise comparison of total exercise dose between the two groups. Second, the participants cannot be blinded due to the nature of the intervention, which may introduce performance bias. Third, the reliance on subjective patient-reported outcomes, such as the SPADI and VAS, rather than objective measures such as isokinetic testing or strength testing, limits the depth of functional assessment. Fourth, the absence of magnetic resonance imaging precludes other shoulder pathologies, such as labral lesions. Fifth, the use of rescue medications (e.g., NSAIDs or acetaminophen) was not quantitatively recorded. Nevertheless, the randomized controlled design of this study likely serves to equilibrate these unmeasured confounding factors between the groups. Lastly, though symptom recurrence was anticipated four weeks after injection, this twelve-week study period may be insufficient for monitoring recurrence. However, this study provided valuable information on the application of remote physiotherapy for chronic subacromial bursitis with impingement syndrome. Future research should incorporate more objective outcome measures, imaging modalities, and longer follow-up for a more comprehensive understanding of the interventions’ effects.

## 5. Conclusions

A mHealth-supported home-based resistance exercise program supported by mHealth significantly extends the benefits of corticosteroid injections in patients with chronic subacromial bursitis compared to education on unsupervised exercises alone.

## Figures and Tables

**Table 1:** Demographic variables of the participants.

Variable	UCI * + RE ^†^ (*n* = 27), Mean (SD)	UCI * (*n* = 26), Mean (SD)	*p* Value
Age (years)	53.6 (9.2)	57.6 (11.6)	0.162 ^a^
Sex (female/male)	10/17	15/11	0.173 ^b^
BMI	24.4 (2.7)	25.3 (3.3)	0.273 ^a^
Affected (left/right)	9/18	9/17	1.000 ^b^
Duration of symptom (m)	9.2 (2.4)	8.4 (2.2)	0.237 ^a^

* UCI: Ultrasound-guided corticosteroid injection; ^†^ RE: resistance exercise; ^a^ *p* value was calculated using independent *t* test to compare both groups. ^b^ *p* value was calculated using chi-square test to compare both groups.

**Table 2:** Measurements at baseline, 4 weeks and 12 weeks after intervention.

Measurement	UCI * + RE ^†^, *n* = 27, Mean (SD)	UCI *, *n* = 26, Mean (SD)	Mean Difference (95% CI)	Inter-Group Comparison (*p*, *d*) ^a^	Group Effect (*p*, η_p_^2^) ^b^	Time Effect (*p*, η_p_^2^) ^b^	Interaction (*p*, η_p_^2^) ^b^
VAS							
Baseline	6.2 (1.2)	6.3 (0.8)	−0.1 (−0.7, 0.4)	0.666, −0.12	0.497, 0.01	<0.001, 0.77	0.898, 0.002
Week 4	2.9 (1.2)	2.9 (1.2)	−0.1 (−0.7, 0.6)	0.827, −0.06			
Week 12	3.4 (1.0)	3.7 (1.3)	−0.3 (−0.9, 0.4)	0.449, −0.21			
SPADI							
Baseline	54.0 (8.5)	55.0 (10.0)	−0.9 (−6.1, 4.2)	0.718, −0.10	0.291, 0.02	<0.001, 0.77	0.04, 0.061
Week 4	30.8 (10.1)	30.0 (10.1)	0.8 (−4.8, 6.4)	0.77, 0.08			
Week 12	31.5 (7.2)	37.2 (8.6)	−6.1 (−10.5, −1.7)	0.007, −0.77			

* UCI: Ultrasound-guided corticosteroid injection; ^†^ RE: resistance exercise; ^a^ Inter-group comparisons were performed using post hoc Tukey’s tests. ^b^ Derived from two-way ANOVA.

**Table 3:** Measurements of range of motion at baseline, 4 weeks and 12 weeks after intervention.

Measurement	UCI * + RE ^†^, *n* = 27, Mean (SD)	UCI *, *n* = 26, Mean (SD)	Mean Difference (95% CI)	Inter-group Comparison (*p*, *d*) ^a^	Group Effect (*p*, η_p_^2^) ^b^	Time Effect (*p*, η_p_^2^) ^b^	Interaction (*p*, η_p_^2^) ^b^
Flexion							
Baseline	144.5 (9.6)	142.8 (10.6)	1.7 (−3.9, 7.3)	0.550, 0.17	0.322, 0.02	<0.001, 0.43	0.032, 0.065
Week 4	155.8 (9.3)	158.1 (5.8)	−2.3 (−6.6, 1.9)	0.283, −0.3			
Week 12	152.8 (10.0)	147.1 (6.5)	5.7 (1.1, 10.3)	0.017, −0.68			
Abduction							
Baseline	137.3 (12.7)	136.3 (14.1)	1.00 (−6.4, 8.4)	0.788, 0.07	0.527, 0.01	<0.001, 0.53	0.036, 0.063
Week 4	153.1 (12.2)	156.5 (7.8)	−3.5 (−9.1, 2.2)	0.227, −0.34			
Week 12	156.7 (6.4)	150.4 (8.5)	6.3 (2.1, 10.4)	0.004, 0.84			
External rotation							
Baseline	56.9 (10.1)	55.4 (11.0)	1.5 (−4.4, 7.3)	0.616, 0.14	0.768, 0.002	0.008, 0.09	0.328, 0.022
Week 4	59.1 (5.7)	60.8 (5.2)	−1.7 (−4.7, 1.3)	0.266, −0.31			
Week 12	59.6 (5.5)	58.1 (4.7)	1.6 (−1.3, 4.4)	0.277, 0.30			
Internal rotation							
Baseline	44.4 (8.5)	43.9 (9.8)	0.6 (−4.5, 5.7)	0.813, 0.007	0.498, 0.01	<0.001, 0.28	0.494, 0.01
Week 4	50.4 (5.5)	51.0 (5.3)	−0.6 (−3.6, 2.4)	0.693, −0.11			
Week 12	52.6 (5.1)	50.2 (5.2)	2.4 (−0.4, 5.2)	0.095, 0.47			

* UCI: Ultrasound-guided corticosteroid injection; ^†^ RE: resistance exercise; ^a^ Inter-group comparisons were performed using post hoc Tukey’s tests. ^b^ Derived from two-way ANOVA.

## Data Availability

The data associated with the paper are not publicly available but are available from the corresponding author on reasonable request.

## Abbreviations

The following abbreviations are used in this manuscript:

BMI	body mass index
mHealth	mobile health
RE	resistance exercise
ROM	range of motion
SPADI	Shoulder Pain and Disability Index
UCI	Ultrasound-guided corticosteroid injection
VAS	Visual Analogue Scale
